# Insights from the Active Use of Neuroscience Findings in Teaching and Learning

**DOI:** 10.3390/bs14080639

**Published:** 2024-07-25

**Authors:** Ausra Daugirdiene, Jurate Cesnaviciene, Agne Brandisauskiene

**Affiliations:** 1 Education Research Institute, Education Academy, Vytautas Magnus University, K. Donelaičio Str. 52, LT-44244 Kaunas, Lithuania; agne.brandisauskiene@vdu.lt; 2 Institute of Psychology, Vilnius University, Universiteto St. 9, LT-01513 Vilnius, Lithuania; 3 Teacher Training Institute, Education Academy, Vytautas Magnus University, K. Donelaičio Str. 52, LT-44244 Kaunas, Lithuania; jurate.cesnaviciene@vdu.lt

**Keywords:** neuroscience, neurodidactics, teaching strategies, learning strategies

## Abstract

The aim of this paper is to show how teachers apply teaching and learning strategies related to the principles of the nervous system’s functions. In our view, understanding what constitutes good teaching is about identifying how it engages the underlying cognitive and neurosystemic processes within the human brain in relation to learning. Using a student self-assessment questionnaire, we have investigated several key processes involved in neurodidactics (excitation, perception, memory, and the use, transfer, and adaptation of information and/or actions). The sample consisted of 884 7–10th grade students. The results showed that students’ excitation, understanding, and consolidation of educational material are directly related to the work of the teacher and the teaching strategies they apply to attract and stimulate the student’s attention and to help the student to understand and remember information. The learning strategies used by the students reflect the learner’s learning activity, i.e., the use and application of strategies that allow internal knowledge to emerge. The consolidation of the learning material and the learning strategies used by the students was statistically significantly higher among the female participants. There are significant differences between low- and high-achieving students in terms of the effectiveness of teaching strategies for consolidation and the learning strategies applied by learners. The paper provides practical recommendations for teachers.

## 1. Introduction

One of the main concerns for educators is how to boost students’ engagement in the learning process and help them achieve higher learning outcomes. Researchers are, therefore, looking at how to create the best possible conditions for students to learn. One direction is the integration of neuroscience with educational sciences [1], combining the collaboration of different researchers (educators, psychologists, neuroscientists) [2]. However, with regard to the relevance of the field of educational neuroscience, a debate is brewing in the research community about whether neuroscience can improve teaching and learning processes in the classroom. For example, Bowers [3] points out that neuroscience rarely adds new insights into learning because psychology has already achieved this. However, an argument from the opposite camp might be Dubinsky et al.’s [4] assertion that although educational neuroscience does not invent new pedagogies, this knowledge can help teachers to make good decisions when teaching students. Neuroscience influences psychological theory, which in turn shapes educational practice [5]. So, if neuro and educational scientists collaborate as equal stakeholders, the prospects for neuroscience-based learning practices will increase [6].

Neuroscience provides explanations about teaching and learning processes [7,8,9], but according to Mayer [2], these are still rarely applied in teaching practice. Teachers are still guided by various neuromyths [10,11,12]. According to a systematic review [13], the three most common neuromyths are related to the beliefs that learners learn better when they receive information in their preferred learning style (e.g., auditory, visual, kinesthetic), that short co-ordination exercises can improve the integration of the functions of the right and left hemispheres, and that we only use 10% of our brains when we learn. It is, therefore, necessary to emphasize that learning is the result of brain activity, a neuro-phenomenon that can be analyzed by answering questions such as: what is learning; how does learning take place; what factors can learning facilitate; and what are the consequences of learning [14]? All of these questions are very important, but this article aims to focus on the teacher dimension of this work by explaining what teacher actions facilitate student learning. The aim of this paper is to show how teachers apply teaching and learning strategies related to the principles of the nervous system functioning. The research is based on theoretical insights, the core of which is neuroactivation, perception, consolidation, and the use, transfer, and adaptation of information and/or actions by students in the learning process as fundamental elements of good teaching and successful learning.

## 2. Theoretical Framework

Learning and teaching is experience based. Experience leads to the growth of interconnected networks of simple cells distributed across the entire brain, which eventually results in complex cognitive structures. Learning is distributed across large networks of neurons, and, according to Goswani [1], the learning environments created in schools by teachers may have important cumulative effects.

Another key issue is active learning, which implies improved outcomes when compared to passive learning. Markant, Ruggeri, Gureckis, and Xu [15] argue that the concept of active learning has grown to encompass a huge variety of instructional techniques, usually referring to a combination of increased physical activity or interaction, deeper processing, elaboration or explanation of material, planning of learning activities, question asking, metacognitive monitoring, and social collaboration.

In our opinion, understanding good teaching requires identifying how it engages underlying cognitive and neurosystem processes within the brain related to learning. We are aware of some basic processes related to neurodidactic activity:

- Excitation can happen when the teacher tells interesting science stories; stimulates expression of joy, wonder, and other positive emotions using their voice, body language, or mimes; includes humorous stories and different forms of humor; uses unexpected analogies and metaphors; and incorporate active work and movement into the lesson. Various teaching and/or learning methods involving student attention and emotions are suitable [16,17,18]. We would like to emphasize that it is not only humorous stories that are important, but also different scientific experiences—suspenseful and unsuccessful—that stimulate student engagement and excitation.
- Perception is the reflection of the totality of an object, event, process, or phenomenon in the psyche by activating or irritating the sensory organs. The result of perception is the percept. The particularity of the perception of every learner is defined by numerous active stimuli, from which the ones that affect the learner’s alertness are selected and perceived. Three stages of perception are undoubtedly significant: attention towards/the selection of information, the structuring of information, and understanding information (interpretation of information) [19].
- Memory is a mental process, including the memorization, preservation, and remembering of information and/or actions. During this process, it is very important that perceived and memorized information is transferred from the sensory memory into the short-term memory; therefore, it is necessary to maintain learners’ attention [20,21]. It is clear that information held in short-term memory can be quickly forgotten if not captured (recorded) in the long-term memory [22,23]. Therefore, individual internal naming and active repetition of information are important for memorizing information. The memory process can also be greatly assisted by a learner’s individual internal language, which is unique and meaningful only to themselves [24]. It is usually used as a thinking tool for solving tasks/problems in difficult circumstances. It is also worth mentioning that our memory is associative and knowledge is better captured by associating it with prior knowledge, namely the information that is already stored in our long-term memory. Association is a prerequisite for learning since it is the formation of a relationship or connection between concepts, images, ideas, and actions in the human nervous system (psyche). Such associations are formed through the acquisition of experience, i.e., through learning and free spontaneous or purposeful reproduction, for example, through knowledge testing, tests, etc. [25,26].
- The use, transfer, and adaptation of information and/or actions at the appropriate time. This process occurs if the teacher allows the information or activity learned to be repeated. Such stimulation to reproduce information may occur immediately after interpretation (immediate) or sometime after memorization (delayed). Reproduction can also be encouraged with intent and willpower (voluntary) or without them (involuntary). We would like to point out that teachers can also promote high-level voluntary memory (information recovery) of students [27]. It is advisable for teachers and students to use a variety of playback techniques as the processes of memorization and reproduction are not identical in time or manner, i.e., something memorized at one time and in a certain way, and reproduced at another time and in another way (for example, visualization of a seen image, picture, diagram, pattern by narrative; or vice versa: reproduction of a narrative by drawing, diagram, map, mind mapping, data or concept notation, timelines, images). Reproduction techniques will be effective if the memorized information is reproduced by linking it to the previously memorized information, and each student will rearrange it in their own way, i.e., will try to shape their inner knowledge [28].

**Figure 1:**
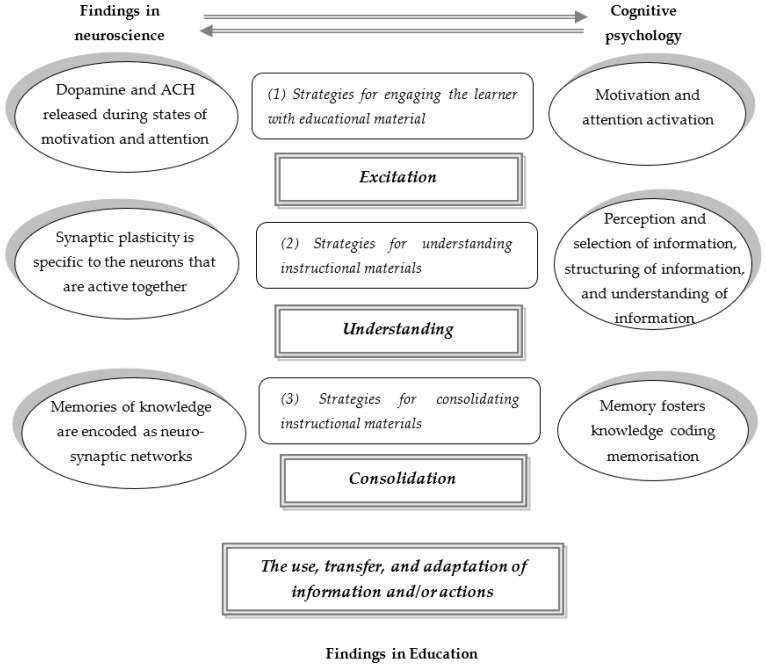
Findings from neuroscience, cognitive psychology, and education.

In Figure 1 below, we linked the findings that are crucial to teaching and learning processes to neuroscience, cognitive psychology, and the educational sciences. Strategies actively applied by teachers (white rectangles), which are based on neuro-didactic principles, trigger the learners’ brain processes (gray rectangles). These processes lead to actively manifested (cognitive) changes in the human cognitive system (ovals on the right of the figure). Meanwhile, the latter (human cognition) are determined by the principles of the functioning of the nervous system (ovals on the left). In our opinion, all of these stages are important to achieving the best possible learning outcomes.

Hence, the teacher can apply the following:

(1) Strategies for excitation the learner with educational material;
(2) Strategies for understanding instructional materials;
(3) Strategies for consolidation of instructional materials.

The importance of the strategies presented in both teaching and learning should be emphasized. By teaching and applying these strategies, the teacher initiates or stimulates the learners’ brain processes that affect cognition. However, the learner also needs to be active as perception, memory processes, use, and transfer of information and/or actions are not possible without the learner’s own involvement. Consequently, both the teacher and the learner must be active. According to Markant, Ruggeri, Gureckis, and Xu [15], for example, one of the brain processes—enhanced memory—may be the common effect of activities (physical interaction, self-pacing, metacognitive monitoring, and goal-driven exploration). Bearing the aforesaid in mind, we have also included the scale “Learning strategies applied by the students” in the instrument we developed.

## 3. Materials and Methods

### 3.1. Participants and Procedure

The survey was carried out in spring 2020. The survey was carried out according to the Declaration of Helsinki and the Code of Ethics for Researchers, and was approved by the Lithuanian Academy of Sciences (Resolution of the LMA Presidium No 22 of 19 June 2012). Written consent from the parents or guardians was obtained before students participated in the survey. The survey is based on respect for the student’s free choice to participate in the study. All students were assured that the survey was voluntary and anonymous. Therefore, the usual ethical procedures, including the confidentiality of the answers, were ensured during the study.

The participants chosen for this study were 7th–10th grade (13–17 years old) students from different schools in Lithuania. The representativeness of the sample was assured by cluster sampling. The research sample involved 884 students. Sample distribution according to gender and grade appears in Table 1.

### 3.2. Measures

A self-report student questionnaire was used. The first section of the research concerned the socio-demographic data (gender, grade), school type (basic school, high school), and two questions about academic grades: “What was your final grade in Mathematics?” and “What was your final grade in Lithuanian language and literature?”. The second section consisted of a questionnaire with 32 items and was designed by the first and third authors. The title of the questionnaire was Teaching and Learning Strategies based on Neuroscience. The items in the questionnaire use a 5-point Likert scale ranging from 1 (almost never) to 5 (almost always) with an intermediate score of 3 (sometimes). The scores in the Teaching and Learning Strategies based on Neuroscience questionnaire were calculated by averaging the individual item scores. The internal consistency of Teaching and Learning Strategies based on Neuroscience questionnaire was assessed using Cronbach’s alpha and McDonald’s omega (Table 2). It was determined that the Cronbach’s alpha (0.932) and McDonald’s omega (0.934) for the present study were both above 0.70, which is an acceptable level of reliability in educational research [29].

The construct validity of the Teaching and Learning Strategies based on Neuroscience questionnaire was assessed using exploratory factor analysis (EFA) and confirmatory factor analysis (CFA). The research sample (n = 884) was randomly split in half to create two separate data sets, one for EFA and one for CFA. The data set for the EFA was made up of a sample of 430 students (Subsample 1). The CFA data set comprised 454 students (Subsample 2). EFA was employed to ascertain the factor structure of the Teaching and Learning Strategies based on Neuroscience questionnaire. The KMO test for MSA was 0.944 and Bartlett’s Test of Sphericity was significant (χ^2^ = 5853.918, df = 496, *p* < 0.0001) indicating that the size of the data set and Subsample 1 was adequate for EFA. A total of four factors were extracted from the factor analysis, accounting for 54.09% of the total variance. To verify the results of the EFA, CFA was performed on Subsample 2. The results of the CFA confirm the structure revealed in the EFA. Fit indices values were found to be: χ^2^/df = 3.59; RMSEA = 0.076 (90% CI = 0.072–0.080); SRMR = 0.071; GFI = 0.972; CFI = 0.935.

### 3.3. Data Analysis

The data were analyzed using IBM SPSS Statistics 26 and IBM SPSS Amos 26. The internal consistency of the questionnaire was tested by the calculation of Cronbach’s alpha and McDonald’s omega. Coefficients values of 0.7 or higher indicate acceptable internal consistency for each scale and for the questionnaire as a whole [29,30]. The construct validity was assessed using exploratory factor analysis (EFA) and confirmatory factor analysis (CFA). EFA were conducted using the principal component analysis (PCA) extraction method, followed by an Oblimin rotation (with Kaiser Normalization). The Kaiser–Meyer–Olkin measure of sampling adequacy (KMO) and Bartlett’s test were used to test if the data were suitable for the factor analysis. KMO test scores should be greater than 0.70, and Bartlett’s test of sphericity must be statistically significant. The number of factors was determined by eigenvalues >1.0 [30]. For CFA, the goodness-of-fit of model was assessed using the following parameters: χ^2^/df ≤5.00, RMSEA ≤0.08, SRMR ≤0.08, CFI ≥0.90, GFI ≥0.90 [31].

For descriptive purposes, the data were analyzed using frequencies, percentages, modes, means, and standard deviations. Skewness and kurtosis were used for normality assessment. For sample size >300, normality of the depends on the absolute values of skewness and kurtosis. Either an absolute skewness value ≤2 or an absolute kurtosis ≤4 may be used as reference values for determining considerable normality [30].

Between-gender differences at four scales were assessed by Student’s *t*-tests. Differences between the 7th-grade, 8th-grade, 9th-grade, and 10th-grade students were determined by ANOVA. For each test, an effect size was calculated to indicate the objective significance of the difference. Cohen’s d (standardized effect size) was calculated to measure the difference between the means of the two groups. For Cohen’s *d*, a value of 0.20–0.40 is interpreted as a small effect, 0.50–0.70 is a medium effect, and 0.80–1.0 ≤ is a large effect. Partial eta-squared (η_p_^2^) was calculated to measure the effect size of the difference between 7th and 10th grades. To interpret the magnitude of effect sizes, η_p_^2^ = 0.01 indicates a small effect, η_p_^2^ = 0.06 indicates a medium effect, η_p_^2^ = 0.14 indicates a large effect [32]. Statistical significance was set at *p* < 0.05.

## 4. Results

### 4.1. Descriptive Statistics

Analyzing the descriptive statistics of the scales presented in Table 3, it appears that teachers in grades 7–10 focus more on the application of teaching strategies for consolidating (M = 3.56, Mo = 3.57) and teaching strategies for understanding (M = 3.10, Mo = 3.14) the instructional material. Teaching strategies for excitation with educational material (activate brain activity) are less frequently used during lessons (M = 2.87, Mo = 2.78) based on the students’ responses.

An analysis of the skewness and kurtosis values indicated that the research data were close to a normal distribution. Therefore, parametric statistics were used for all analyses.

### 4.2. Differences in Four Scales

Student’s *t*-test was used to compare the means of the four scales between the boys and girls (Table 4). The obtained results reveal that the means of two scales—teaching strategies for consolidation and learning strategies applied by the student—in the sample of the girls is statistically significantly higher than that of the boys (*p* < 0.0001). While the Student’s *t*-test showed statistically significant differences, the Cohen’s d (d*_Cohen_* = 0.3) indicates that the effect was small.

Table 5 presents the differences in the means of the scales by grade. For the means of three scales—teaching strategies for understanding, teaching strategies for consolidation, learning strategies applied by the students—there was no statistically significant difference between the groups. However, the ANOVA with post hoc Tukey’s tests showed that teachers tend to employ excitation strategies (such as learning new material, using games, telling short funny stories related to educational material) more often when teaching 9th-grade students (M = 2.98) than 7th-grade students (M = 2.80) (F = 2.810; *p* < 0.05). Although this difference is statistically significant, the effect is small (η_p_^2^ = 0.01).

We also compared two student groups: students with low and high achievement. There are two significant differences between these groups (Table 6 and Table 7). Students with higher achievement in Lithuanian language and literature have a higher mean score (M = 3.66) on the teaching strategies for consolidations scale than students with lower achievement (M = 3.40). This difference is statistically significant (*p* < 0.0001). Despite reaching statistical significance, the mean difference was quite small (d*_Cohen_* = 0.04). The mean for the learning strategies applied by students scale (M = 3.43) is also higher for those students with higher achievement (*p* < 0.0001). In this case, the effect size is medium (d*_Cohen_* = 0.06).

To summarize the results of the study (Table 7), it was found that students with higher mathematics achievement had higher mean scores on the teaching strategies for consolidation and learning strategies applied by students scales (respectively, M = 3.69 and M = 3.36) than students with lower achievement (respectively, M = 3.47 and M = 3.05). Student’s *t*-test showed that the differences were statistically significant (*p* < 0.0001), but it was a small difference (d*_Cohen,_* respectively, 0.03 and 0.04).

## 5. Discussion

Our empirical study aimed to see how teachers apply teaching strategies related to the principles of nervous system functioning. Before discussing the results, we would like to point out that the data we have collected are based on students’ opinions only, and we acknowledge that this as a limitation of this work. It is clear that there is a need for further research and objective data collection through classroom observation, teacher interviews, and experimental design. Nevertheless, some trends are evident, and we present them below.

First of all, Lithuanian teachers do not tend to use strategies based on neuroscience knowledge very actively, i.e., the results for all four scales are mediocre (the mode ranges from 2.78 to 3.57). This finding can be explained by the fact that teachers do not have enough knowledge related to neuroscience. In Lithuania, only in recent years have pre-service teachers and educational support specialists received training in neuroscience. We would venture to say that this lack of knowledge is not unique to teachers in our country (Lithuania). As mentioned above, neuromyths are still used by teachers in various countries [10,11,12,13].

Second, the results of the study demonstrate that the teachers used strategies to activate brain activity (neuro-stimulation) least frequently; namely the teaching strategies for excitation scale has the lowest mean score. From a neurodidactic perspective, brain activation strategies are very important in lessons because they primarily stimulate (activate) the learner and encourage concentration. Focusing attention is the first principle of a good memory. Good memorization can occur when students focus a greater degree of attention on the material they want to learn. This means that students are more likely to engage in learning when teachers present the material well, i.e., the material stimulates their brain activity. As is known, the brain functions more actively when there are innovations in the environment, for example, new or unknown stimuli, because students notice them almost immediately and try to understand what they are and think about them. As the authors of [33,34] state, if students in the classroom lack impressions and excitement, they need neurostimulation. The brain adapts and responds actively only when it faces a new, different, or other situation [35]; therefore, neurostimulation strategies used by teachers are significant.

It is interesting to note that the results of our study show that the teachers tend to employ teaching strategies for excitation (such as learning new material, using games, telling short funny stories et, etc., related to educational material) more often when teaching older (9th-grade students) rather than younger (7th-grade) students (this is the only difference when comparing the results by student class). This seems somewhat unexpected, but can be explained by the essence of the teaching strategies used for neurostimulation and/or excitation. Teachers should attract and focus students’ attention, but they do not cover the active cognitive activities of the student. A student’s excitation alone will not necessarily be linked to learning outcomes as the complete learning process includes excitation, understanding, and consolidation of learning material. Younger students may like the excitement of a fun neurostimulation activity, but they may not use other teaching strategies as effectively and may be easily distracted by the variety of tasks. Meanwhile, older pupils may easily switch to other teaching strategies when their interest is aroused, which is why, in our opinion, teachers are more likely to intuitively use teaching strategies for excitation in older classes.

According to the participants in the study, teachers use Teaching strategies for understanding materials slightly more actively than Teaching strategies for excitation. This means that teachers question students during the teaching process in order to understand the extent to which students have grasped the material. When applying strategies to understand the learning material, the three aforementioned stages of perception take place: the selection of information (we again refer to attention), the structuring of information, and the understanding of information (interpretation of information). Thus, it is important to concentrate when new information is received. We would also like to stress a positive learning experience, for example, finding a good, correct solution to the task increases the amount of dopamine in the brain, which in turn leads to positive feelings (joy) [36,37,38]. The student will be willing to repeat this experience, which means that a positive learning experience presupposes a desire to learn and can lead to good learning outcomes; therefore, the work of an educator requires creating conditions for a successful learning experience [39].

The results showed that teaching strategies for consolidation are the most intensively used. It is related to the active and continuous use of the information obtained. By repeating and practicing, i.e., ‘working’ with a certain material, students remember it better because they create and strengthen the connections of neurons that transmit that information. Our assumption as to why teachers are more likely to use this type of strategy is that reinforcement is associated with active teaching methods that seek to engage the student in the learning process. Lithuanian students’ achievement is not high [40], so teachers probably intuitively focus more on reinforcing certain subject knowledge and skills.

Thirdly, when comparing the survey data by gender, there is some difference. The obtained results reveal that the means of two scales—teaching strategies for consolidation and learning strategies applied by the students—in the sample of the girls is statistically significantly higher than that of the boys. Perhaps this result can be explained by girls’ higher motivation to learn. It is likely that they are more likely to have a higher level of aspiration for higher learning achievement, and therefore, pay more attention to the teacher’s reinforcement tasks and use more active learning strategies at home. However, it should be noted that although a statistically significant gender difference was found, the effect was small.

Finally, it turns out that students with higher achievement in Lithuanian language and literature and mathematics have statistically significantly higher mean scores on two of the scales (teaching strategies for consolidation and learning strategies applied by students) than their lower-achieving peers. Our obtained results are probable, because if students have accumulated more information (as demonstrated by higher averages on the teaching strategies for consolidation scale) and are more active in their independent learning (with higher averages on the learning strategies applied by students scale), then they will also score higher in these subjects. Meaningful use of learning strategies helps the student to take control of their own learning process, while increasing their confidence and motivation to learn. Many researchers point to this link. For example, they argue that the learning strategies used by students are also significant for the effective learning process of students [41,42]. During the past few decades, research on students’ learning and achievement has progressively put more emphasis on cognitive strategies, metacognition, motivation, and task engagement [43]. These are student actions that include a deep understanding of learning material, linking it to existing prior knowledge, and meaningful practice of new subject material (knowledge, skills) [44].

## 6. Conclusions

One of the areas of research that is needed is studies that link the field of education with neuroscience and draw increasingly clearer links between how teaching affects learning and our understanding of how the nervous system and brain work. This is a completely new field for educators in our country. The results of the study show that teachers sometimes use teaching strategies related to the functioning of the nervous system, but that they do so in a less proactive and, presumably, intuitive way. It is, therefore, clear that a teacher should not be limited to such intuitive teaching. Professional knowledge that includes the most relevant knowledge of today’s neuroscience, psychological, and educational sciences is essential. As Rodriguez [45] argues, only highly skilled teachers with systematic thinking who recognize the different parts of the pedagogical work (the student, the teacher and how they interact) in the system, and who know how they interact and influence each other, can create effective learning environments in which all learners can succeed. It is the teacher’s awareness that is essential for teaching ability and student achievement [46]. Therefore, from our point of view, it is essential that teachers acquire knowledge related to the teaching and learning process based on the integration of the above-mentioned sciences. An active and engaged learner is essential for a successful learning process, and up-to-date knowledge of all the sciences related to the learning process is, therefore, essential for every teacher.

## 7. Practical Implications and Future Research

Practical recommendations for teachers from this work include the following: (1) All teachers should receive professional development on the principles of the nervous system. This would help them to understand what didactic methods should be used in the classroom and what is the neurophysiological basis of these methods; (2) all four of the neurostrategies we have mentioned should be applied in the classroom, but none of them should overshadow or dominate the others; (3) teachers should encourage all students, regardless of their academic achievement, to apply learning strategies applied by students as this builds students’ autonomy and confidence and teaches them to learn actively and effectively.

It should also be noted that it is necessary to continue the investigations that have been launched. Research that combines knowledge from education, psychology and neuroscience is of great importance for educational practice. Longitudinal studies that help to achieve learning success for each student could be exceptional here.

## Figures and Tables

**Table 1:** Sample characteristics.

	Boys		Girls		Total	
	n	%	n	%	n	%
7th grade	145	33.8	155	34.1	300	33.9
8th grade	124	28.9	133	29.2	257	29.1
9th grade	67	15.6	88	19.3	155	17.5
10th grade	93	21.7	79	17.4	172	19.5
Total	429	100	455	100	884	100

**Table 2:** Internal consistency in the Teaching and Learning Strategies based on Neuroscience questionnaire scales.

Scales	Cronbach α	McDonalds ω	Number of Items	Sample of Items
Teaching strategies for excitation	0.857	0.863	9	Teachers tell short, entertaining stories related to educational materials.
Teaching strategies for understanding	0.815	0.815	7	Teachers encourage me to explain in my own words what I have understood.
Teaching strategies for consolidation	0.782	0.774	7	Teachers present different tasks in the same lesson (e.g., you have to write or to speak).
Learning strategies applied by the students	0.862	0.866	9	As I study the material, I find similarities and differences between the phenomena.

**Table 3:** Descriptive statistics.

Scales	Mode	Mean	SD	Skewness	Kurtosis
Teaching strategies for excitation	2.78	2.87	0.71	0.119	0.116
Teaching strategies for understanding	3.14	3.10	0.75	−0.131	0.033
Teaching strategies for consolidation	3.57	3.56	0.68	−0.691	1.118
Learning strategies applied by students	3.44	3.20	0.76	−0.337	0.234

**Table 4:** Differences in four scales between genders.

Scales		Mean	SD	Student’s *t*-Test		d*_Cohen_*
				t	*p*	
Teaching strategies for excitation	Boys	2.85	0.69	−1.072	0.284	-
	Girls	2.90	0.72			
Teaching strategies for understanding	Boys	3.03	0.73	−1.094	0.274	-
	Girls	3.08	0.77			
Teaching strategies for consolidation	Boys	3.45	0.70	−4.623	0.0001	0.3
	Girls	3.66	0.66			
Learning strategies applied by students	Boys	3.07	0.76	−4.933	0.0001	0.3
	Girls	3.32	0.74			

**Table 5:** Differences in four scales among 7th–10th-grade students.

Scales		Mean	SD	ANOVA Test		η_p_^2^
				F	*p*	
Teaching strategies for excitation	7th grade	2.80	0.72	2.810	0.039	0.01
	8th grade	2.87	0.72			
	9th grade	2.98	0.64			
	10th grade	2.92	0.73			
Teaching strategies for understanding	7th grade	3.04	0.76	1.601	0.188	-
	8th grade	3.03	0.78			
	9th grade	3.17	0.71			
	10th grade	3.00	0.72			
Teaching strategies for consolidation	7th grade	3.49	0.73	1.455	0.225	-
	8th grade	3.60	0.68			
	9th grade	3.57	0.66			
	10th grade	3.60	0.62			
Learning strategies applied by students	7th grade	3.17	0.79	0.645	0.586	-
	8th grade	3.24	0.76			
	9th grade	3.24	0.75			
	10th grade	3.17	0.72			

**Table 6:** Differences in four scales according to performance level in Lithuanian language and literature.

Scales	Performance Level	Mean	SD	Student’s *t*-Test		d*_Cohen_*
				t	*p*	
Teaching strategies for excitation	Low achievement	2.85	0.73	−0.398	0.691	-
	High achievement	2.88	0.75			
Teaching strategies for understanding	Low achievement	3.06	0.77	0.243	0.808	-
	High achievement	3.05	0.80			
Teaching strategies for consolidation	Low achievement	3.40	0.74	−3.806	0.0001	0.4
	High achievement	3.66	0.70			
Learning strategies applied by students	Low achievement	2.98	0.78	−6.364	0.0001	0.6
	High achievement	3.43	0.72			

**Table 7:** Differences in four scales according to performance level in mathematics.

Scales	Performance Level	Mean	SD	Student’s *t*-Test		d*_Cohen_*
				t	*p*	
Teaching strategies for excitation	Low achievement	2.85	0.72	−0.853	0.394	-
	High achievement	2.90	0.65			
Teaching strategies for understanding	Low achievement	3.06	0.73	−0.141	0.888	-
	High achievement	3.06	0.72			
Teaching strategies for consolidation	Low achievement	3.47	0.69	−3.796	0.0001	0.3
	High achievement	3.69	0.63			
Learning strategies applied by students	Low achievement	3.05	0.75	−4.744	0.0001	0.4
	High achievement	3.36	0.73			

## Data Availability

The datasets generated and analyzed during the current study are not publicly available due to privacy and ethical concerns but are available from the corresponding author on reasonable request.

## References

[1] Goswani U. (2008). Principles of learning, implications for teaching: A cognitive neuroscience perspective. J. Philos. Educ..

[2] Mayer R.E. (2017). How can brain research inform academic learning and instruction?. Educ. Psychol. Rev..

[3] Bowers J.S. (2016). The practical and principled problems with educational neuroscience. Psychol. Rev..

[4] Dubinsky J.M., Roehrig G., Varma S. (2022). A place for neuroscience in teacher knowledge and education. Mind Brain Educ..

[5] Thomas M.S.C., Ansari D., Knowland V.C.P. (2019). Annual research review: Educational neuroscience: Progress and prospects. J. Child Psychol. Psychiatr..

[6] Edelenbosch R., Kupper F., Krabbendam L., Broerse J.E.V. (2015). Brain-based learning and educational neuroscience: Boundary work. Mind Brain Educ..

[7] Fischer K.W. (2009). Mind, brain, and education: Building a scientific groundwork for learning and teaching. Mind Brain Educ..

[8] Howard-Jones P., Scott A., Gordillo C. (2024). The science of microteaching and learning: An exploratory study. Mind Brain Educ..

[9] Rodriguez V. (2013). The human nervous system: A framework for teaching and the teaching brain. Mind Brain Educ..

[10] Arslan Y., Gordon R., Tolmie A. (2022). Teachers’ understanding of neuromyths: A role for educational neuroscience in teacher training. Impact.

[11] Gini S., Knowland V., Thomas M.S.C., Van Herwegen J. (2021). Neuromyths about neurodevelopmental disorders: Misconceptions by educators and the general public. Mind Brain Educ..

[12] Macdonald K., Germine L., Anderson A., Christodoulou J., McGrath L.M. (2017). Dispelling the myth: Training in education or neuroscience decreases but does not eliminate beliefs in neuromyths. Front. Psychol..

[13] Torrijos-Muelas M., González-Víllora S., Bodoque-Osma A.R. (2021). The Persistence of Neuromyths in the Educational Settings: A Systematic Review. Front. Psychol..

[14] Donoghue G.M., Horvath J.C. (2016). Translating neuroscience, psychology and education: An abstracted conceptual framework for the learning sciences. Cogent Educ..

[15] Markant D.B., Ruggeri A., Gureckis T.M., Xu F. (2019). Enhanced memory as a common effect of active learning. Mind Brain Educ..

[16] LeDoux J.E. (1996). The Emotional Brain.

[17] Kovalik S., Olsen K.D. (1998). How emotions run us, our students, and our classrooms. NASSP Bull..

[18] Zusho A., Kumar R., Bondie R.S. (2023). Transforming fear into rigor, love, freedom, and joy: A new paradigm of standards-based reform. Educ. Psych..

[19] Goldstein E.B., Cacciamani L. (2021). Sensation and Perception.

[20] Engle R.W. (2002). Working memory capacity as executive attention. Curr. Dir. Psychol. Sci..

[21] Engle R.W. (2018). Working memory and executive attention: A revisit. Perspect Psychol. Sci..

[22] Engle R.W., Tuholski S.W., Laughlin J.E., Conway A.R.A. (1999). Working memory, short-term memory, and general fluid intelligence: A latent-variable approach. J. Exp. Psychol. Gen..

[23] Finn A.S., Sheridan M.A., Hudson Kam C.L., Hinshaw S., D’Esposito M. (2010). Longitudinal evidence for functional specialization of the neural circuit supporting working memory in the human brain. J. Neurosci..

[24] Finn A.S., Minas J.E., Leonard J.A., Mackey A.P., Salvatore J., Goetz C., West M.R., Gabrieli C.F.O., Gabrieli J.D.E. (2017). Functional brain organization of working memory in adolescents varies in relation to family income and academic achievement. Dev. Sci..

[25] Ackerman P.L., Woltz D.J. (1994). Determinants of learning and performance in an associative memory/substitution task: Task constraints, individual differences, volition, and motivation. J. Educ. Psychol..

[26] Yang C., Luo L., Vadillo M.A., Yu R., Shanks D.R. (2021). Testing (quizzing) boosts classroom learning: A systematic and meta-analytic review. Psychol. Bull..

[27] Fazio L.K., Agarwal P.K., Marsh E.J., Roediger H.L. (2010). Memorial consequences of multiple-choice testing on immediate and delayed tests. Mem. Cognit..

[28] Hunt R.R., Worthen J.B. (2006). Distinctiveness and Memory.

[29] DeVellis R.F., Thorpe C.T. (2021). Scale Development: Theory and Applications.

[30] Watkins M.W. (2021). A Step-by-Step Guide to Exploratory Factor Analysis with SPSS.

[31] Brown T.A. (2015). Confirmatory Factor Analysis for Applied Research.

[32] Cohen L., Manion L., Morrison K. (2018). Research Methods in Education.

[33] Lipina S.J., Evers K. (2017). Neuroscience of childhood poverty: Evidence of impacts and mechanisms as vehicles of dialog with ethics. Front. Psychol..

[34] Rosen M.L., Sheridan M.A., Sambrook K.A., Meltzoff A.N., McLaughlin K.A. (2018). Socioeconomic disparities in academic achievement: A multi-modal investigation of neural mechanisms in children and adolescents. NeuroImage.

[35] McGinty J., Radin J., Kaminski K. (2013). Brain-friendly teaching supports learning transfer. New Dir. Adult Contin. Educ..

[36] Braver T.S., Brown J.W. (2003). Principles of pleasure prediction: Specifying the neural dynamics of human reward learning. Neuron.

[37] Harley C.W. (2004). Norepinephrine and dopamine as learning signals. Neural Plast..

[38] Duncan K., Shohamy D., Kahana M.J., Wagner A.D. (2022). Dopamine and Learning. The Oxford Handbook of Human Memory.

[39] Dunlosky J., Rawson K.A., Marsh E.J., Nathan M.J., Willingham D.T. (2013). Improving students’ learning with effective learning techniques: Promising directions from cognitive and educational psychology. Psychol. Sci. Public Interest.

[40] OECD (2019). PISA 2018 Results (Volume I): What Students Know and Can Do.

[41] Rosário P., Núñez J.C., Vallejo G., Cunha J., Azevedo R., Pereira R., Nunes A.R., Fuentes S., Moreira T. (2016). Promoting gypsy children school engagement: A story-tool project to enhance self-regulated learning. Contemp. Educ. Psychol..

[42] Vandevelde S., Van Keer H., Merchie E. (2017). The challenge of promoting self-regulated learning among primary school children with a low socioeconomic and immigrant background. J. Educ. Res..

[43] Paris S.G., Paris A.H. (2001). Classroom applications of research on self-regulated learning. Educ. Psychol..

[44] Doyle T.J., Doyle B.M., Hoidn S., Klemenčič M. (2020). Learning and teaching in harmony with the brain. sights from neuroscience, biology, cognitive science and psychology. In The Routledge International Handbook of Student-Centered Learning and Teaching in Higher Education.

[45] Rodriguez V. (2013). The Potential of systems thinking in teacher reform as theorized for the teaching brain framework. Mind Brain Educ..

[46] Rodriguez V., Solis S.L. (2013). Teachers’ awareness of the learner–teacher interaction: Preliminary communication of a study investigating the teaching brain. Mind Brain Educ..

